# Innovation in the treatment, interventions and systems of care for opioid use disorder: opportunities to understand multimorbidities

**DOI:** 10.1192/j.eurpsy.2024.124

**Published:** 2024-08-27

**Authors:** A. M. Baldacchino

**Affiliations:** Medicine, University of St Andrews, St Andrews, United Kingdom

## Abstract

**Introduction:**

Opioid use disorder is still the main presenting illicit substance use disorder that patients present within Addiction and Mental Health Services even though the majority of the patients are polysubstance users. Innovation in the field will allow providers to understand better how systems work to support a population with physical and psychological morbidities

**Method:**

We will present novel narratives in describing:

1. Standards and principles
2. Pharmacology
3. Delivery systems
4. Neuroscience based interventions
5. Systems and implementation

**Results and Discussion:**

The above desscriptors will allow a landscape that is less stigmatising and better in responding to the needs of the people who are highly stigmatised and multidisdvantaged.

**Disclosure of Interest:**

None Declared

